# Decreased Openness to Experience Is Associated with Migraine-Type Headaches in Subjects with Lifetime Depression

**DOI:** 10.3389/fneur.2017.00270

**Published:** 2017-06-22

**Authors:** Mate Magyar, Xenia Gonda, Dorottya Pap, Andrea Edes, Attila Galambos, Daniel Baksa, Natalia Kocsel, Edina Szabo, Gyorgy Bagdy, Rebecca Elliott, Gyongyi Kokonyei, Gabriella Juhasz

**Affiliations:** ^1^MTA-SE-NAP B Genetic Brain Imaging Migraine Research Group, Hungarian Academy of Sciences, Semmelweis University, Budapest, Hungary; ^2^Department of Neurology, Faculty of Medicine, Semmelweis University, Budapest, Hungary; ^3^MTA-SE Neuropsychopharmacology and Neurochemistry Research Group, Hungarian Academy of Sciences, Semmelweis University, Budapest, Hungary; ^4^Department of Psychiatry and Psychotherapy, Semmelweis University, Budapest, Hungary; ^5^Faculty of Education and Psychology, Institute of Psychology, Eötvös Loránd University, Budapest, Hungary; ^6^Department of Pharmacodynamics, Faculty of Pharmacy, Semmelweis University, Budapest, Hungary; ^7^Neuroscience and Psychiatry Unit, The University of Manchester, Manchester, United Kingdom; ^8^Manchester Academic Health Sciences Centre, Manchester, United Kingdom

**Keywords:** migraine, major depressive disorder, personality traits, neuroticism, openness, extraversion

## Abstract

**Introduction:**

Migraine and depression frequently occur as comorbid conditions, and it has been hypothesized that migraine with and without depression may have a different genetic background. A distinct personality trait constellation has been described in migraineurs. Less attention, however, was paid to personality differences in migraineurs with and without depression which may also shed light on differences in the neurobiological, background. The aim of our study was to investigate big five personality traits, headaches, and lifetime depression (DEP) in a large European general population sample.

**Methods:**

Relationship between DEP, Big Five Inventory personality traits, and headaches identified by the ID-Migraine Questionnaire were investigated in 3,026 individuals from Budapest and Manchester with multivariate and logistic regression analyses.

**Results:**

Both DEP and migraine(ID) showed differences in personality traits. Neuroticism was an independent risk factor for both conditions while a significant interaction effect appeared between the two in the case of openness. Namely, subjects with migraine(ID) and without DEP scored higher on openness compared to those who had depression.

**Conclusion:**

While we confirmed previous results that high neuroticism is a risk factor for both depression and migraine, openness to experience was significantly lower in the co-occurrence of migraine and depression. Our results suggest that increased openness, possibly manifested in optimal or advantageous cognitive processing of pain experience in migraine may decrease the risk of co-occurrence of depression and migraine and thus may provide valuable insight for newer prevention and intervention approaches in the treatment of these conditions.

## Introduction

Migraine is a neurovascular disorder presenting with recurrent episodes of often unilateral, moderate to severe headache attacks associated with nausea, vomiting, phono-, and/or photophobia (1). Migraine is one of the most common pain disorders (2), affecting approximately 11% of the general population (3), with patients reporting lower productivity and quality of life than the healthy population (3, 4). In addition, migraine frequently co-occur with other neuropsychiatric disorders; one of the most frequent comorbidities in migraine is depression. Approximately half of major depressive disorder patients also report coexisting severe headache, in most cases migraine (5, 6), which is a much higher prevalence than in the general population (4, 7). Based on the results of prospective studies, migraine and major depression mutually increase the risk of each other suggesting a bidirectional relationship between them (4, 7).

The co-occurrence of migraine and depression can be partially explained by shared genetic risk factors (8), but the exact mechanism of this co-occurrence is not well understood (6). Genetic studies indicate that migraine and anxious depression are partly influenced by overlapping genetic and non-shared environmental factors (9). However, the relationship between migraine and depression is complex and a causative pathway has yet to be established. Nevertheless, the fact that same biological pathways, e.g., the serotonergic and dopaminergic systems, affect anxiety, depression, and migraine (10, 11) suggests that disturbances of these systems may increase the risk of one or more of these disorders (9).

Clinically, pain can be considered as a symptom of depression, supported by various observations showing that not only migraine but also other pain symptoms and syndromes show a higher prevalence in depressed patients (12, 13). Conversely, risk of depression may be increased by recurring migraine headaches (4). Comparing symptom profiles of migraineurs with and without major depression reveals only minor differences; the prevalence of aura symptoms and the aggravation of migraine by physical activity being higher in severe migraineurs with major depression compared to those without depression (5). In addition, allodynia was more prevalent in those with anxiety and depression symptoms (14). Thus, a similar overall migraine symptom profile suggests that a similar disease process may play a role in both groups. Characteristics of depression, however, show a more marked difference between migraineurs and non-migraineur depressives, with a significantly higher number of depressive episodes as well as a significantly higher prevalence of seasonal variation, irritability, and affective temperaments observed in those with comorbid migraine and major depression (15). Migraine has been found to show a particularly high prevalence in bipolar II disorder, and clinical features of those unipolar patients who also have co-occurring migraine resemble bipolar II patients suggesting important clinical differences between migraineur and non-migraineur unipolar depressives (15, 16).

Besides minor differences in symptom profiles in depressed and non-depressed migraineurs, and the more characteristic and also clinically relevant differences between migraineur and non-migraineur depressives, an important question is whether there is a distinct personality profile in those with co-occurring migraine and depression as compared to those with only migraine or only depression or with neither condition. Neuroticism or emotional lability has been found to be independently associated with both migraine (17–19) and major depression (20, 21) sharing a significant portion of genetic risk, suggesting that neuroticism may be a shared risk factor for depression and migraine. However, only a subset of depressive and migraineur patients manifest the comorbidity of these disorders which indicates that, in spite of the overlapping genetic and other risk factors, there may be important factors which protect from the comorbid development of migraine and depression. Studies show that extraversion (16) and openness, agreeableness, and conscientiousness (16, 22) may play a protective role in the development of migraine. Similarly, higher levels of conscientiousness, agreeableness, and extraversion were found to be protective against depression (23). Further understanding of such personality factors in increasing and decreasing risk of migraine or depression and understanding its relationship to their co-occurrence would be crucial not only to better understand the neurochemical and biopsychosocial contributors of migraine but also to help identify targets for prevention and intervention at both biological and psychological levels.

The aim of our present study was to investigate possible personality trait differences in those who suffered from migraine-type headache in the last 3 months with and without lifetime depression (DEP) in a large average population cohort recruited in Budapest and Manchester. Based on previous studies, we hypothesized that beside neuroticism, which is a common risk factor for migraine(ID) and DEP, we can identify personality factors that are less prevailing in the case of comorbidity of migraine(ID) and DEP. In addition, we investigated our hypothesis in sufferers of non-migraine headaches and other pain disorders to test migraine specificity.

## Materials and Methods

### Subjects

The present study was part of an EU funded research programme, called NewMood (New Molecules in mood Disorders), which aimed to investigate novel patho-mechanisms of major depression and its comorbid disorders, such as anxiety and migraine (24). Details of the recruitment and responses can be found elsewhere (25, 26). To shortly summarize, participants were recruited in Greater Manchester, UK and Budapest, Hungary by contacting general practices and using advertisements (NewMood website, university advertisements, and newspapers). All willing participants filled out the NewMood booklet, which contained brief standard and validated questionnaires. Altogether *n* = 2,004 subjects responded at Manchester and *n* = 1,139 at Budapest by sending back the postal questionnaire and the signed written informed consent. From them, subjects with useful questionnaire data were included in this study regardless of ethnicity or reported medical or psychiatric disorders. In Manchester, data from 1,970 participants with a mean age of 33.50 years, and in Budapest, data from 1,056 participants with a mean age of 31.40 years, were analyzed. The study was approved by local ethics committees and was carried out in accordance with the Declaration of Helsinki.

### Questionnaires

Background details (e.g., sex, age), information on socioeconomic status, and medical history, including psychiatric disorders and reported migraine, were collected by a brief standard background questionnaire, English and Hungarian versions, respectively.

Reported DEP episodes were derived from a set of questions that were validated in a subset of participants during face-to-face interviews (27). Other pain disorders were determined based on the background questionnaire and coded “yes” if subjects did not report migraine but reported at least one of these conditions: back pain (*n* = 162), rheumatoid arthritis (*n* = 31), abdominal pain (*n* = 43, e.g., irritable bowel syndrome, Crohn’s disease, ulcerative colitis, heartburn), joint pain (*n* = 16, e.g., arthritis, osteoarthritis), diffuse pain (*n* = 14, e.g., fibromyalgia, myalgic encephalomyelitis, complex regional pain), or other pain (*n* = 4, e.g., mastitis, chronic sinusitis).

To identify subjects with migraine(ID) type headache, the ID-migraine questionnaire was used, which is a validated screening tool for migraine (28, 29). Migraine(ID) was coded if the participants experienced at least two symptoms out of nausea, photophobia, and disability during headaches in the previous 3 months (28). Non-migraine headache was coded if only one symptom was experienced, or the subject indicated that they had headaches without these symptoms and therefore not fulfilling the criteria for migraine(ID).

The Big Five Inventory was used to measure five factors of personality (BFI-44), namely, extraversion, agreeableness, conscientiousness, neuroticism, and openness (30). Items are rated on a 5-point Likert-type scale ranging from 1 (disagree strongly) to 5 (agree strongly). For personality factors, continuous weighted dimension scores (sum of item scores divided by the number of items completed) were calculated for the analysis.

### Statistical Analysis

SPSS 21.0 for Windows (IBM) was used to carry out statistical analyses. Pearson chi^2^ analysis calculated the difference of migraine(ID), non-migraine headaches, and pain prevalence in subjects with or without DEP. Multivariate ANOVA in the whole population (Budapest and Manchester together) was applied to investigate whether there was an interaction effect of DEP and migraine(ID) on personality factors. Wilk’s lambda statistics and test of between-subject effects were reported. Using univariate ANOVA, *post hoc* we calculated the significant interaction effects in the subpopulations separately to identify replicable findings, and also tested whether the significant interaction was migraine specific or applied to non-migraine headaches or other pain. Age and sex were covariates in all analyses. In calculations where the whole population was included, study site was added as an independent factor to the analysis to control for cohort differences. Finally, a logistic regression model was built to test the effect of age, sex, the five personality factors, DEP, and any significant interaction between DEP and personality on migraine(ID). All statistical testing adopted a two-tailed *p* = 0.05 threshold Table 1.

**Table 1:** Characteristics of the investigated populations.

	Total population	Manchester	Budapest
**Demographics**			
Participant number (*n*)	3,026	1,970 (65%)	1,056 (35%)
Female (*n*, %)	2,082 (69%)	1,341 (68%)	741 (70%)
Age (mean SE)	32.8 (0.19)	33.5 (0.23)	31.4 (0.33)
**Migraine, headaches, and pain**			
Migraine(ID) (*n*, %)	829 (27%)	586 (30%)	243 (23%)
Proportion of Migraine(ID) without/with lifetime depression (DEP) (*n*, %)	353 (20%)/476 (38%)	190 (20%)/396 (39%)	163 (20%)/80 (35%)
Non-migraine headache (*n*, %)	1,380 (46%)	838 (43%)	542 (51%)
Other pain disorders	239 (8%)	139 (7%)	100 (10%)
**Psychometric measures**			
Reported DEP (*n*, %)	1,246 (41%)	1,016 (52%)	230 (22%)
BFI neuroticism (mean SE)	3.15 (0.02)	3.32 (0.02)	2.83 (0.03)
BFI extraversion (mean SE)	3.29 (0.02)	3.15 (0.02)	3.55 (0.03)
BFI conscientiousness (mean SE)	3.67 (0.01)	3.65 (0.02)	3.70 (0.02)
BFI agreeableness (mean SE)	3.76 (0.01)	3.75 (0.01)	3.78 (0.02)
BFI openness (mean SE)	3.74 (0.01)	3.63 (0.01)	3.94 (0.02)

*ID, data derived from the ID-Migraine questionnaire (28); BFI, Big Five Inventory (30)*.

## Results

The demographic characteristics of the investigated populations are displayed in Table 1.

Similar to the scientific literature, subjects with DEP reported significantly more migraine(ID) (DEP: 38% vs. no-DEP: 20%, Pearson chi^2^ = 124.4, df = 1, *p* < 0.001), more other pain disorders (DEP: 10% vs. no-DEP: 7%, Pearson chi^2^ = 11.3, df = 1 *p* = 0.001), but less non-migraine headaches (DEP: 40% vs. no-DEP: 50%, Pearson chi^2^ = 27.9, df = 1, *p* < 0.001).

**Figure 1:**
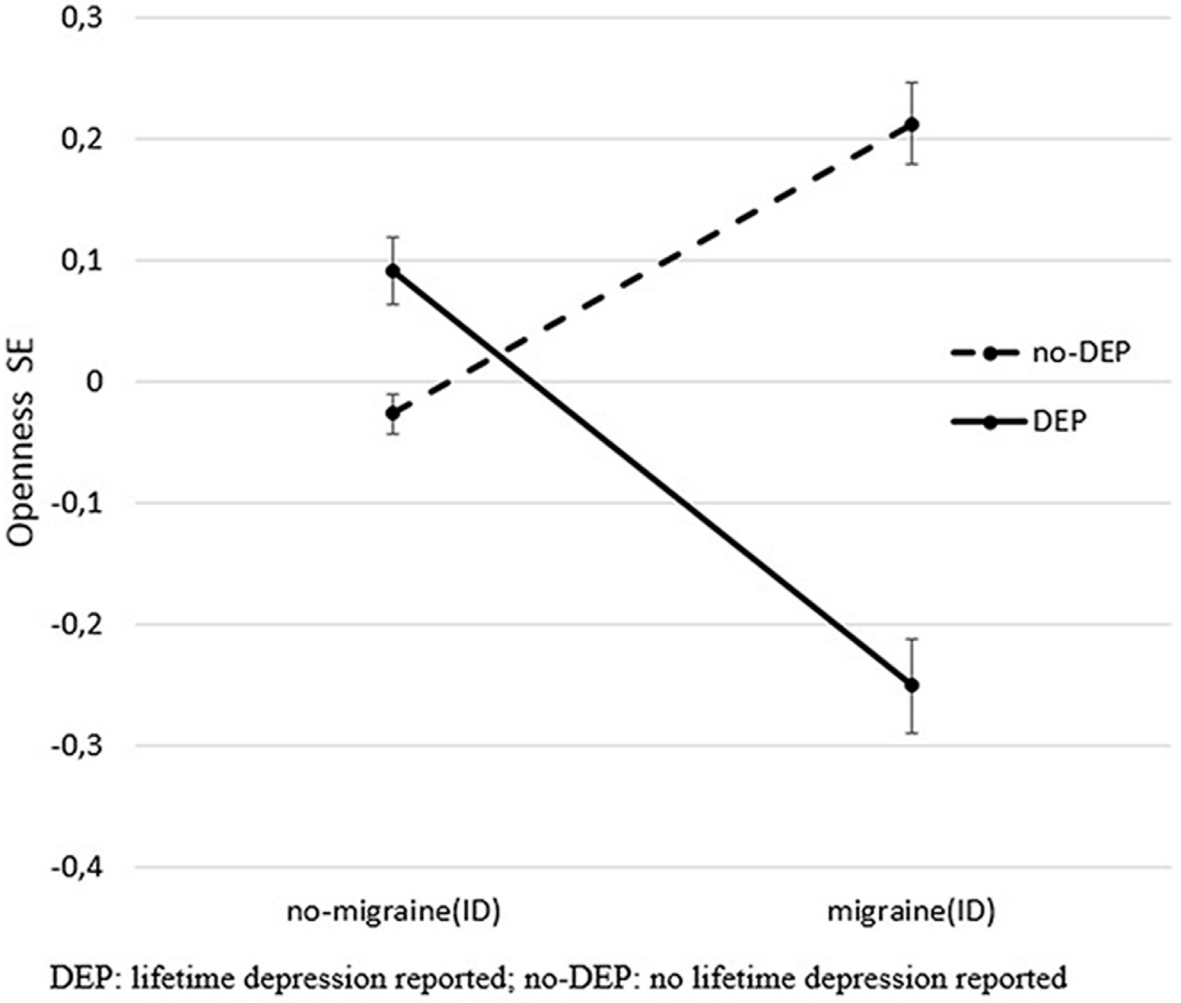
Significant interaction effect of DEP and migraine(ID) on openness. The figure shows standardized openness to experience scores (Big Five Inventory) and SEM. DEP, lifetime depression; no-DEP, no lifetime depression.

After controlling for age, sex, and study site, results of MANOVA indicated main effects of both DEP and migraine(ID), and their interaction effect on personality dimensions (Table 2, B). According to the test of between-subject, effect indicated that DEP and non-DEP subjects differed in extraversion, agreeableness, contentiousness, and neuroticism significantly. Subjects with or without migraine(ID) significantly diverged on extraversion, agreeableness, and neuroticism. Only one interaction emerged between DEP and Migraine(ID), namely, on openness (Figure 1). *Post hoc* univariate ANOVA demonstrated significant DEP and migraine(ID) interaction on openness in both the Budapest (*F* = 6.467, df = 1, 1,050 *p* = 0.011) and Manchester cohort (*F* = 4.759, df = 1, 1,970, *p* = 0.029). Openness to experience scores were lower in DEP + migraine(ID) individuals compared to individuals without migraine(ID) and/or DEP.

**Table 2:** MANOVA on personality factors to investigate the effect of DEP and migraine(ID) in the whole population.

(A) Multivariate test Wilks’ lambda indicated that DEP and migraine significantly interact with personality factors							

Effect		*F*		df		Sig	
Intercept		*3,008.795*		*5, 3,012*		*<0.001*	
Sex		*67.179*		*5, 3,012*		*<0.001*	
Age		*35.914*		*5, 3,012*		*<0.001*	
DEP		*72.458*		*5, 3,012*		*<0.001*	
Migraine(ID)		*12.999*		*5, 3,012*		*<0.001*	
Cohort		*37.163*		*5, 3,012*		*<0.001*	
DEP × Migraine(ID)		*3.213*		*5, 3,012*		*0.007*	
DEP × cohort		*8.080*		*5, 3,012*		*<0.001*	
Migraine(ID) × cohort		0.425		5, 3,012		0.831	
DEP × Migraine(ID) × cohort		0.682		5, 3,012		0.637	

**(B) Univariate ANOVA ***post hoc*** results separately on the five personality factors in tests where MANOVA Wilks’ lambda were significant**							

**Effect**	**Personality factor**	**Whole population**		**Manchester**		**Budapest**	
		***F***	**Sig**	***F***	**Sig**	***F***	**Sig**

Sex	Extraversion	*11.240*	*0.001*	*11.579*	*0.001*	0.982	0.322
	Agreeableness	*60.904*	*<0.001*	*43.036*	*<0.001*	*18.521*	*<0.001*
	Conscientiousness	*37.718*	*<0.001*	*24.583*	*<0.001*	*13.446*	*<0.001*
	Neuroticism	*80.292*	*<0.001*	*48.447*	*<0.001*	*31.707*	*<0.001*
	Openness	*19.421*	*<0.001*	*34.230*	*<0.001*	0.279	0.597
Age	Extraversion	*6.689*	*0.010*	2.237	0.135	*5.415*	*0.020*
	Agreeableness	*42.611*	*<0.001*	*42.056*	*<0.001*	*4.690*	*0.031*
	Conscientiousness	*130.075*	*<0.001*	*94.455*	*<0.001*	*36.904*	*<0.001*
	Neuroticism	*13.461*	*<0.001*	*12.200*	*<0.001*	2.255	0.133
	Openness	*5.265*	*0.022*	*4.430*	*0.035*	1.175	0.279
DEP	Extraversion	*77.908*	*<0.001*	*82.655*	*<0.001*	*22.569*	*<0.001*
	Agreeableness	*37.760*	*<0.001*	*25.970*	*<0.001*	*18.186*	*<0.001*
	Conscientiousness	*73.671*	*<0.001*	*61.861*	*<0.001*	*27.943*	*<0.001*
	Neuroticism	*341.452*	*<0.001*	*498.703*	*<0.001*	*58.369*	*<0.001*
	Openness	2.232	0.135	1.097	0.295	1.120	0.290
Migraine(ID)	Extraversion	*11.452*	*0.001*	*5.573*	*0.018*	*6.356*	*0.012*
	Agreeableness	*7.507*	*0.006*	*4.285*	*0.039*	3.715	0.054
	Conscientiousness	1.515	0.219	1.868	0.172	0.289	0.591
	Neuroticism	*62.933*	*<0.001*	*41.385*	*<0.001*	*27.375*	*<0.001*
	Openness	0.572	0.450	0.111	0.739	1.533	0.216
Cohort	Extraversion	*43.509*	*<0.001*				
	Agreeableness	0.810	0.368				
	Conscientiousness	0.299	0.585				
	Neuroticism	*73.189*	*<0.001*				
	Openness	*103.784*	*<0.001*				
DEP × Migraine(ID)	Extraversion	0.005	0.945	0.080	0.777	0.068	0.795
	Agreeableness	2.264	0.133	*5.092*	*0.024*	0.120	0.729
	Conscientiousness	0.000	0.991	0.368	0.544	0.191	0.662
	Neuroticism	1.651	0.199	0.854	0.356	0.883	0.348
	Openness	*10.653*	*0.001*	*4.759*	*0.029*	*6.467*	*0.011*
DEP × cohort	Extraversion	1.070	0.301				
	Agreeableness	0.471	0.493				
	Conscientiousness	0.000	0.987				
	Neuroticism	*29.631*	*<0.001*				
	Openness	0.001	0.981				

*DEP, lifetime depression; cohort, Budapest vs. Manchester*.

*Significant results in italics*.

**Figure 2:**
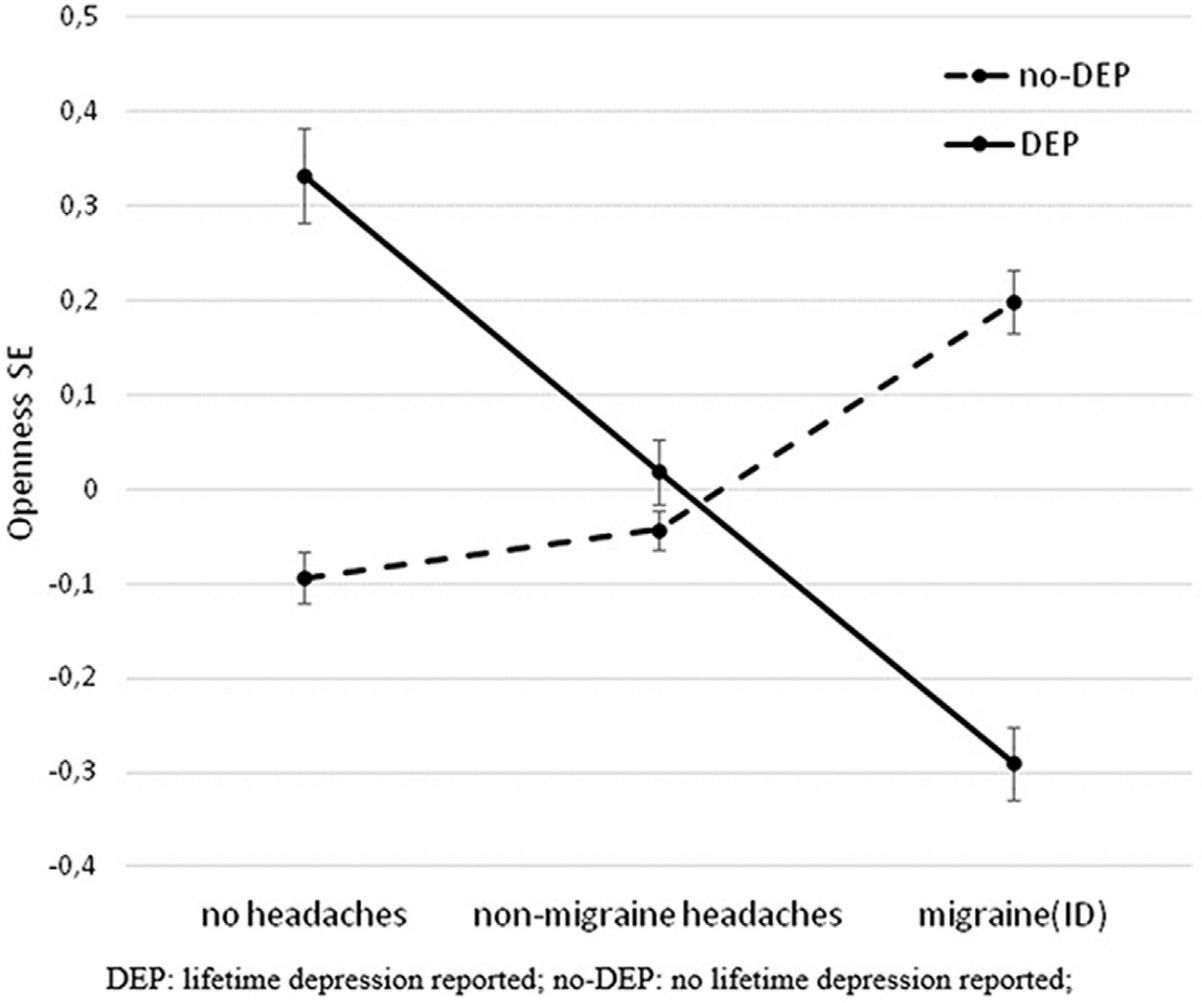
Significant interaction effect of DEP and headaches on openness in the total population. The figure shows standardized openness to experience scores (Big Five Inventory) and SEM. DEP, lifetime depression; no-DEP, no lifetime depression.

Categorizing headaches into non-migraine headaches and migraine(ID), a similar interaction effect was demonstrated between DEP and headaches on openness (*F* = 6.107, df = 2, 3,012, *p* = 0.002, Figure 2) in the whole study population (corrected for age, sex, and study site). The difference between DEP and non-DEP subjects was significant in those who had no headaches in the past 3 months (*F* = 3.867, df = 1, 811, *p* = 0.05) and in the migraine(ID) group (*F* = 7.160, df = 1, 823, *p* = 0.008). No significant difference emerged in the non-migraine headaches group (*F* = 0.392, df = 1, 1,374, *p* = 0.532). Furthermore, there was no significant interaction between DEP and other pain disorders (*F* = 0.490, df = 1, 3,016, *p* = 0.484) on openness.

Logistic regression analysis showed that by taking into account sex, age, cohort, personality factors, DEP, and DEP by openness interaction, four variables increased the risk of migraine(ID), namely, sex, neuroticism, openness, and DEP. Besides, DEP by openness interaction still significantly decreased the odds ratio for migraine(ID) after controlling for all the above variables (Table 3). Furthermore, contrary to previous results, our data also demonstrated that openness to experience was increased in subjects with migraine(ID) but higher openness was only present in those migraineurs who did not suffer from DEP. Thus, openness to experience may prevent the co-occurrence of migraine and depression. Interestingly, we could not demonstrate a similar effect in the case of non-migraine headaches, or other pain disorders suggesting that openness to experience might represent a specific protective mechanism toward the comorbidity of depression and migraine.

**Table 3:** Logistic regression on migraine(ID) adding sex, age, cohort, personality factors, DEP, and DEP by openness interaction.

Variables	OR	95% CI for OR		Wald	df	Sig.
		Lower	Upper			
Sex	*1.95*	*1.58*	*2.42*	*37.55*	*1*	*<0.001*
Age	1.00	0.99	1.00	1.12	1	0.290
Cohort	1.03	0.84	1.25	0.07	1	0.796
Extraversion	0.97	0.87	1.09	0.22	1	0.642
Agreeableness	1.02	0.88	1.19	0.10	1	0.753
Contentiousness	1.05	0.92	1.19	0.54	1	0.463
Neuroticism	*1.68*	*1.47*	*1.91*	*60.49*	*1*	*<0.001*
Openness	*1.32*	*1.07*	*1.62*	*6.93*	*1*	*0.008*
DEP	*4.95*	*1.79*	*13.68*	*9.52*	*1*	*0.002*
DEP by openness	*0.73*	*0.56*	*0.95*	*5.49*	*1*	*0.019*
Constant	*0.01*			*51.43*	*1*	*<0.001*

*OR, odds ratio; CI, confidence interval; DEP, lifetime depression*.

*Significant results in italics*.

## Discussion

In line with the previous literature, our results in a large European sample further supported that migraine and depression are frequently comorbid conditions, and both DEP and neuroticism independently increase the risk of reporting migraine-type headaches. Furthermore, contrary to previous results, our data also demonstrated that openness to experience is an independent risk factor for migraine(ID) but higher openness is only present in those migraineurs who do not suffer from DEP. Thus, openness to experience may prevent the co-occurrence of migraine and depression. Interestingly, we could not demonstrate similar protective effect in the case of other or mixed headaches, or other pain disorders suggesting that openness to experience might represent a specific protective mechanism toward depression in migraine.

### Openness to Experience in Health and Diseases

We found important associations between personality traits and both migraine and depression. As previous studies suggested (19, 20), neuroticism appears as a risk factor for both migraine(ID) and DEP in our population study. However, in contrast to previous studies (16, 22), we found that openness increased the risk of migraine, but we also found an important interaction effect in case of openness, indicating higher openness only in migraine(ID) patients without depression.

Openness manifested in a characteristic constellation of cognitive and affective styles, including creativity, curiosity, flexible thinking, preference of novel experience, increased receptiveness for salient stimuli, and absorption in sensory experience (31, 32). Although this is the most controversial trait in the 5-factor model (33, 34), recent studies indicated that it is an important factor in coping with different disorders. Indeed, openness has been reported to play a protective role against depression in longitudinal follow-up studies (35). Similarly, openness was associated with reduced rates of depression among people with somatic conditions such as among hemodialysis patients (36) or in postpartum period (37), also indicating that openness may help to counteract the depressogenic effects of somatic conditions. In addition, openness has been found to be associated with lesser physiological reactivity and higher physiological adaptation, measured by hart rate, blood pressure, and respiratory rhythm changes, to recurrent social evaluative stress, suggesting that openness might be a protective factor against harmful effects of stress (38). Regarding migraine, in line with our results, migraineurs with higher openness are also more flexible and creative in their approach to managing their condition which in turn reduces the impact of migraine on their daily life, which is also reflected in less impaired function in migraineurs with higher openness (39). This effect is especially important in the comorbidity of depression and migraine as migraine and depression have already been reported to have a peculiar relationship reflected by their high comorbidity. In line with our results, this relationship is different from that between depression and other types of headaches (4, 40).

### Specific Relationship between Migraine and Depression

Our study supported previous accounts suggesting a positive association between depression and migraine; however, DEP had an opposite effect on non-migraine headaches. According to a 2-year follow-up study, preexisting depression increased the risk for developing migraine by 3.4 times and preexisting migraine increased risk for developing depression 5.8-fold (4), while current depression and anxiety was associated with a higher increase in the risk of migraine compared to other types of pains and headaches (40). This specific relationship between depression and migraine, but not other types of headaches, may indicate a common genetic or neurobiological element in the pathophysiology of migraine and depression as suggested previously (6, 8).

The nature of the relationship between painful conditions in general and depression is far from well understood and several factors may play a role in the increased co-occurrence specifically of migraine and depression. There may be a direct causal association, although the direction is unclear. Depression may contribute to increased pain sensitivity, or migraine may lead to depression, through recurrent pain and decreased quality of life (9) or through learned helplessness due to its inescapable nature. Depression may also arise as a neurobiological consequence and side effect of migraine-associated pain. A further possibility is that migraine and depression may be different symptomatic manifestations of the same underlying syndrome (9). Recent studies indicated that common pleiotropic genetic and environmental influences may also account for the increased comorbidity between depression and migraine, including the involvement of common biological pathways such as the dopaminergic and serotonergic pathways, and stress as a major risk factor for both conditions (9).

### Possible Mechanism of Openness As a Protective Factor in the Co-Occurrence of Migraine and Depression

Although general heritability of big five traits appear to be around 50% reflecting a strong biological background (41), less neurobiological explanations are available for openness compared to other traits such as neuroticism or extraversion (34). Several authors suggest a higher order solution for the 5-factor model with two superfactors, stability (including neuroticism, agreeableness, and conscientiousness) related to the variation in serotonergic function, and plasticity (including extraversion and openness) related to the variation of dopaminergic function (42–44). A key feature of openness is the exploratory tendency on a more abstract, motivational and cognitive level (34). The dopaminergic system modulates the novelty-associated rewarding positive stimuli (34, 45, 46) and though this possibly regulates both the motivational and cognitive aspects of openness (34).

Based on previous observations, openness appears to be associated with prefrontal and anterior cingulate dopaminergic projections (34, 43, 45). In a recent study, there was a positive association between openness and functional connectivity between the right DLPFC and the right midbrain substantia nigra/ventral tegmental area, which is the chief source of dopaminergic inputs during resting state and different tasks (32). Specific association of dopaminergic regulation in the DLPFC with openness is supported by a robust association between neurocognitive tasks reflecting DLPFC function and openness but not extraversion (34). Thus, DLPFC function could be a key mediator of the protective effect of openness on co-occurrence of migraine and depression, as DLPFC has a particular role in both depression and migraine. Decreased DLPFC function was associated with increased vulnerability to depression in the presence of negative stimuli (47). In addition, the DLPFC was found to be involved in pain modulation (48) and was hypothesized to show a constant upregulation in order to enhance the descending modulation of pain (49, 50).

DLPFC was also implicated to play a role in pain processing specifically in migraine, as greater evoked pain-related activity was reported in interictal migraine patients in several areas related to cognitive pain processing, including the DLPFC (51). Further studies that specifically tested pain–cognition interaction in migraineurs demonstrated that DLPFC showed similar cognitive task related deactivation regardless of pain condition (evoked pain or no pain) showing chronic DLPFC engagement in migraineurs across all conditions (49). In addition, reduced cortical gray matter volume in areas including the DLPFC was associated with pain catastrophizing in migraineurs (52).

Thus, openness in migraine may indicate a more optimal manifestation of enhanced DLPFC activity engaging not only with frontal top-down control to reduce responses to negative stimuli (47), including pain (49, 50), but also flexibly increasing receptiveness for novel salient stimuli and thus contributing to less burden and leading to less depression. Furthermore, higher openness to experience in a healthy sample was related to better default mode network (DMN) efficiency (53) indexing the network integrity. Since both depression (54) and chronic pain conditions (55) have been proved to be associated with alterations in DMN connectivity, we speculate that openness might exert its protective effect against depression *via* better DMN integrity in migraine.

### Therapeutic Consequence of Increased Openness

It has been demonstrated that comorbid depression with other somatic disorders is an independent predictor of unfavorable treatment outcome (56). In the case of migraine, a recent study showed that comorbid depression worsens the responsiveness for acute migraine treatment in a large population sample with episodic migraine (57). Thus, understanding the neurobiological background of this exceptionally high and specific comorbidity between migraine and depression may improve our treatment strategies. In this context, it is interesting to note that openness was associated with increased response to placebo analgesia in migraineurs (58) and with better response to both pharmaco- and psychotherapy in depressed people (59). Furthermore, deep transcranial magnetic stimulation of the DLPFC significantly reduced both frequency and intensity of migraine attacks and depressive symptoms (60).

### Limitations

The study has some limitations. Due to our cross-sectional design, we cannot draw any conclusions about the causal relationship between migraine, depression, and openness. In addition, assessment of both DEP and pain and headache was based on self-report in our study. Using ID-Migraine Questionnaire that covers only the last 3 months increased the risk of including some migraineurs with less frequent, lifelong migraine in the non-migraine group However, it is worth noting that ID-Migraine questionnaire is a valid screening tool for migraine with good specificity and sensitivity (61) and the sensitivity and specificity of the ID-migraine questionnaire in our study was in a comparable range (62) with the original validation study (28) and with the meta-analysis results (61). Also, in our study the prevalence of depression was higher in the Manchester cohort than in the Budapest cohort, despite similar recruitment strategies. However, the proportion of migraine(ID) in the subgroups of subjects with and without DEP were very similar in both cohorts, and the significant interaction of DEP and migraine(ID) on openness replicated in both cohorts. Further studies with clinical samples and longitudinal design are needed to reveal the importance of openness on either daily life or on neural activity.

## Conclusion

In our study investigating the role of personality factors in the co-occurrence of migraine and depression, we found support for the independent risk role of neuroticism and depression in migraine, and also observed decreased openness in case of co-occurrence of these conditions. Our results shed important light on distinguishing features of migraine occurring with and without depression with respect to personality traits. These results may help to understand the biopsychosocial background of migraine and also pave the way of novel strategies in prevention and intervention both on pharmaco- and psychotherapeutic levels to develop personalized treatment approaches.

## Ethics Statement

Both studies were approved by local Ethics Committees (Scientific and Research Ethics Committee of the Medical Research Council, Budapest, Hungary, ad.225/KO/2005; ad.323-60/2005-1018EKU and ad.226/KO/2005; ad.323-61/2005-1018 EKU; North Manchester Local Research Ethics Committee, Manchester, UK REC reference number: 05/Q1406/26) and were carried out in accordance with the Declaration of Helsinki.

## Author Contributions

All persons who meet authorship criteria are listed as authors, and all authors certify that they have participated sufficiently in the work to take public responsibility for the content, including participation in the concept, design, analysis, writing, or revision of the manuscript. Conception and design of study: GB, GJ, XG, and RE. Acquisition of data: MM, XG, GJ, DP, AE, AG, DB, NK, ES, and RE. Analysis and/or interpretation of data: MM, GJ, XG, RE, and GK. Drafting the manuscript: MM, XG, GJ, and GK. Revising the manuscript critically for important intellectual content: MM, XG, DP, AE, AG, DB, NK, ES, GB, RE, GK, and GJ. Approval of the version of the manuscript to be published: MM, XG, DP, AE, AG, DB, NK, ES, GB, RE, GK, and GJ.

## Conflict of Interest Statement

The authors declare that the research was conducted in the absence of any commercial or financial relationships that could be construed as a potential conflict of interest.
